# Phytochemical Profile and Biological Activity of the Ethanol Extracts from the Aerial Parts of *Adonis tianschanica* (Adolf.) Lipsch. Growing in Kazakhstan

**DOI:** 10.3390/molecules29235754

**Published:** 2024-12-05

**Authors:** Saule Orynbekova, Wirginia Kukula-Koch, Zuriyadda Sakipova, Bashaer Alsharif, Beibhinn Rafferty, Talgat Nurgozhin, Zoya Allambergenova, Piotr Dreher, Kazimierz Głowniak, Fabio Boylan

**Affiliations:** 1 School of Pharmacy, S.D. Asfendiyarov Kazakh National Medical University, Almaty 050012, Kazakhstan; sakipova.z@kaznmu.kz (Z.S.); zoyaallambergen@mail.ru (Z.A.); 2 Department of Pharmacognosy with Medicinal Plant Garden, Medical University of Lublin, 1 Chod’zki Street, 20-093 Lublin, Poland; 3 School of Pharmacy and Pharmaceutical Sciences, Panoz Institute, and Trinity Biomedical Sciences Institute, Trinity College Dublin, Dublin 2, D02 PN40 Dublin, Ireland; alsharib@tcd.ie (B.A.); rafferbe@tcd.ie (B.R.); fabio.boylan@tcd.ie (F.B.); 4 Faculty of Pharmacy, Umm Al-Qura University, Makkah 21955, Saudi Arabia; 5 School of General Medicine, Asfendiyarov Kazakh National Medical University, Almaty 050012, Kazakhstan; tnurgozhin@kaznmu.kz; 6 Department of Public Health, Faculty of Medicine, Medical University of Lublin, 20-093 Lublin, Poland; piotr.dreher@lublin.eu; 7 Department of Cosmetology, The University of Information Technology and Management in Rzeszow, Sucharskiego 2, 35-225 Rzeszow, Poland; kglowniak@pharmacognosy.org

**Keywords:** *Adonis tianschanica* (Adolf.) Lipsch., flora of Kazakhstan, HPLC-MS fingerprinting, HSCCC, isoquercitrin, cytotoxic effects, anti-inflammatory activity, NO production

## Abstract

*Adonis tianschanica* is a lesser-known plant species belonging to the genus *Adonis* that grows in Kazakhstan. The aim of this study was to characterize the composition of the ethanolic, water, and hydroethanolic extracts from the aerial parts of *A. tianschanica* by HPLC-ESI-QTOF-MS/MS to isolate the major compound isoquercitrin by HSCCC (High-Speed Counter-Current Chromatography) and to determine the cytotoxicity and anti-inflammatory potential of the extracts produced with this plant. Fingerprinting of the analyzed extracts showed the presence of a multitude of metabolites comprising polyphenols, organic acids, and coumarins, and only trace quantities of cardiac glycosides in the analyzed samples. Flavonoids were certainly the best-represented group, with kaempferol, quercetin, and their derivatives as the major components of the extracts. Key findings in this paper were that the ethanol: water (50:50 *v*/*v*) extract of *A. tianschanica* and its major compound isoquercitrin were able to reduce the production of NO induced by LPS, in addition to demonstrating anti-inflammatory effects by reducing cytokines such as IL-6, TNF-α, and IL-1β.

## 1. Introduction

*Adonis* L. is a genus of plants belonging to the Ranunculaceae family, widely recognized in traditional medicine for its effects on the cardiovascular system. The genus is named after Adonis, a figure in Greek mythology, and the lover of the Goddess Aphrodite (Venus). Native to Europe and Asia, *Adonis* species have also been introduced to North America. They thrive across diverse ecological conditions, ranging from high-altitude regions and continental or Mediterranean semi-deserts to temperate zones. Their distribution spans the western edge of Europe (e.g., the British Isles) to Western and Eastern Siberia, with climates varying from continental and sharply continental to monsoon-influenced regions of the Far East [1,2].

According to the Plant List, the genus *Adonis* comprises 35 species of annual and perennial herbaceous flowering plants. Among these, seven species are recorded in Kazakhstan: *Adonis apennina* L., *Adonis villosa* Ledeb., *Adonis tianschanica* (Adolf.) Lipsch., *Adonis chryzocyathus* Hook. f. et al., *Adonis vernalis* L., *Adonis wolgensis* Siev., and *Adonis aestivalis* L. [3,4].

This genus has garnered significant attention due to its cardiotonic effects. Advances in phytochemistry have led to the identification of numerous bioactive compounds, including more than 50 cardiac glycosides, which are key contributors to its therapeutic potential. Other compounds, such as flavones, carotenoids, and coumarins, have been isolated and exhibit diverse pharmacological properties, including antibacterial, antioxidant, and anti-inflammatory activities. To date, more than 120 distinct chemical compounds have been identified in Adonis species, underscoring their value as sources of medicinal agents [5].

The pharmacological potential of *Adonis*, particularly in cardiovascular medicine, has long been recognized. Early investigations, such as those by Heyl et al. [6], have highlighted the cardiotonic effects of *A. vernalis*, demonstrating its ability to induce systole in frog heart preparations. Subsequent studies have confirmed these findings, showing efficacy comparable to that of digitalis in various bioassays [7,8]. Research has also expanded to other species, such as *A. amurensis*, which enhances cardiac contractility and delays atrioventricular conduction [9], and *A. brevistyla* and *A. pseudoamurensis*, both of which show promise in heart failure treatment [10,11].

In addition to their cardiotonic effects, *Adonis* species exhibit diverse pharmacological properties. For example, anti-inflammatory properties, including inhibition of tumor necrosis factor-α (TNF-α), have been observed in *A. vernalis* extracts [12]. *A. wolgensis* extracts demonstrate antimicrobial efficacy against pathogens such as *Salmonella enteritidis* and *Escherichia coli*. Additionally, antioxidant activities attributable to phenolic compounds are evident in free radical-scavenging assays [13].

*Adonis* plants also show cytotoxic effects against malignant cell lines, with compounds such as amurensiosides and cymarins demonstrating significant activity [14,15]. These antiangiogenic properties [16] suggest potential applications in cancer therapy. Other notable activities include diuretic effects, observed in *A. coerulea* as a traditional Tibetan remedy, and acaricidal properties, further highlighting the genus’s therapeutic versatility [17,18].

These findings establish *Adonis* as a valuable source of bioactive compounds and emphasize the need to further explore its pharmacological potential.

Due to the broad pharmacological activity of *Adonis vernalis* and its long history of use in the treatment of various diseases, exploring other plant species within the same genus is of significant scientific interest. This study aimed to characterize the metabolite profile of the lesser-known species *Adonis tianschanica* (Adolf.) Lipsch, collected from Kazakhstan, isolated the principal constituents and evaluated the pharmacological effects of its aqueous and alcoholic extracts.

*Adonis tianschanica* (Adolf.) Lipsch. is a rhizomatous herbaceous perennial plant that typically reaches a height of up to 35 cm at flowering. It is characterized by branched stems originating from the base and covered with numerous curly hairs, typically ranging from 1 to 5. The leaves are scaly and twice pinnately divided into lanceolate lobes, initially densely hairy at the start of the growing season but becoming nearly glabrous by the time of fruiting. The plant produces solitary flowers, 3.5–5 cm in diameter, with lemon-yellow petals that are slightly irregular in shape. The root system consists of a shortened vertical rhizome, up to 6 cm in length, from which numerous straight, cord-like, dark brown roots extend, reaching up to 30 cm in length; the lateral roots are relatively sparse. The fruit is a polycarp, with seeds measuring 3–4 mm in length and 2–3 mm in width, which are finely wrinkled, scattered with hairs, and feature a small, hook-shaped, downward bent appendage. *A. tianschanica* is found in the northern and central regions of the Tien Shan mountains, extending across Kazakhstan, Kyrgyzstan, and China [19,20,21,22,23].

In this study, we focused on *A. tianschanica*, providing information on the composition of its extracts by HPLC-ESI-QTOF-MS/MS fingerprinting analyses, isolating isoquercitrin, the major compound in the hydroethanolic extract, by HSCCC, measuring its cytotoxic potential, and determining its impact on nitrogen oxide (NO) production and cytokine release. This information will hopefully shed new light on the potential of this underestimated and insufficiently studied plant species, and open new possibilities for its use in traditional Central Asian medicine.

## 2. Results

### 2.1. Compositional Studies of A. tianschanica Extracts by HPLC-MS Fingerprinting

In this study, a thorough fingerprinting of *Adonis tianschanica* (AT) extracts was carried out using high-performance liquid chromatography-electrospray ionization-quadrupole mass spectrometry (HPLC/ESI-QTOF-MS/MS), which is characterized by its high sensitivity and accuracy of mass measurements. The application of high-resolution mass spectrometry in both positive and negative ionization modes, as well as at different fragmentation and collision energies, allowed us to obtain clear chromatograms and informative fragmentation spectra. The identification of the extract components was carried out mainly in the negative ionization mode due to a high diversity of polyphenols that were better detected under these conditions. The positive mode provided information on the presence of cardiac glycosides, although their presence was scarce in the investigated extracts. The table below (Table 1) shows a tentative identification of metabolites in the tested samples. Even if the compound assignment was tentative, it was performed with particular attention using mass databases that included MS/MS fragmentation information (also presented in Table S1 and Figure S1 in the Supplementary File) and the scientific literature on different *Adonis* spp.

As presented in Table 1, the ethanol and water extracts from AT were rich sources of polyphenols. The compounds’ assignment shows a rich representation of kaempferol derivatives, including kaempferol acetylgalactoside, kaempferol sinapoylglucosyl-galactoside, kaempferol glucosyl-glucoside, kaempferol acetylglucoside-glucoside and interesting kaempferol derivatives like hexose-tetrose kaempferol derivatives that seem to be characteristic for AT, but have not yet been assigned in the scientific literature. Besides kaempferol derivatives, the extracts were rich in isoquercitrin, orientin, vitexin, isovitexin, luteolin (with the characteristic adonivernith glucoside), isoorientin, and their glucosides. Certainly, flavonoids are the group of metabolites most represented in the AT extracts analyzed herein. After that, organic acids, organic alcohols, coumarins, alkaloids, fatty acids, and traces of two cardiac glycosides were annotated.

A relative quantitative analysis was performed to identify the best solvent that can be used for the extraction of polyphenols from A. tianschanica. For this purpose, the peak areas of the given metabolites were collected from water, ethanol, and ethanol: water (50:50 *v*/*v*) extracts and compared with one another. The highest peak area of a selected metabolite was assigned as 100%, and the remaining peak areas in the other two solvents were assigned as the percentage content relative to the 100% peak. As shown in Table 2, the water extract contained the smallest amounts of components tentatively identified in the HPLC-MS analysis. The ethanol: water extract (50:50 *v*/*v*) and ethanol extract with greater efficiency recovered glycosides and aglycones of flavonoids. The former extract was the leading one in the recovery of isoquercitrin, isoorientin, isovitexin, and luteolin glucoside. Ethanol extraction resulted in higher yields of kaempferol glucosides and fatty acids. Based on these observations, it can be concluded that ethanol-containing extracts are rich sources of polyphenols. However, 50% EtOH contains a higher diversity of compounds from the plant matrix.

### 2.2. The Fractionation of AT Extract

**Figure 1:**
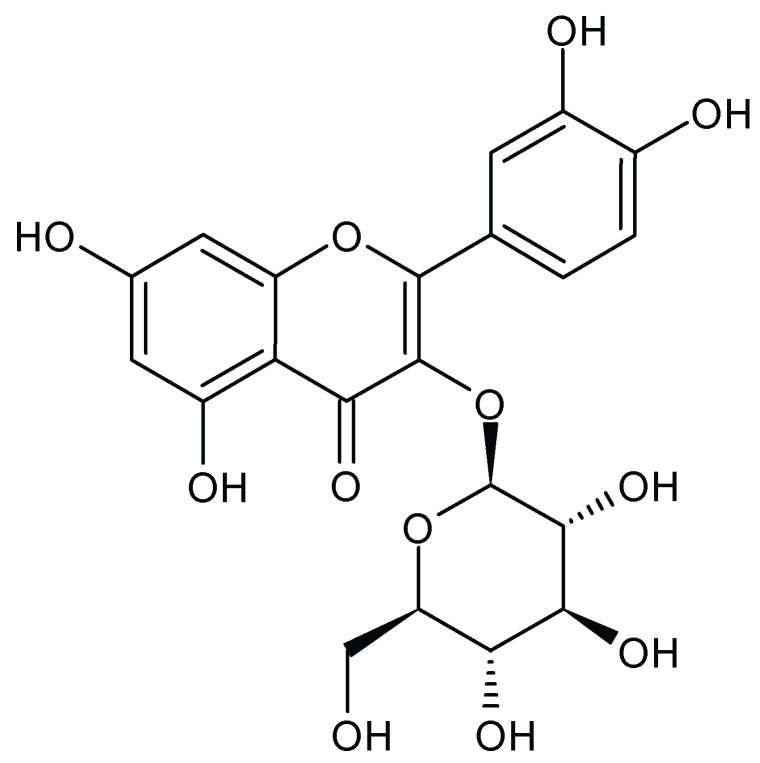
Chemical structure of Isoquercetrin.

HSCCC analysis isolated 80 fractions. TLC analysis using the NP-PEG reagent revealed that several fractions contained flavonoids, as indicated by the presence of yellow bands. One of these fractions was selected for further purification, which yielded the major compound (Figure 1).

Major compound: yellow precipitate; **^1^H-NMR** (400 MHz, DMSO-*D*_6_) δ (ppm): 7.55 (d, *J* = 2.2 Hz, 1H), 7.53 (dd, *J* = 8.5, 2.2 Hz, 1H), 6.80 (d, *J* = 8.5 Hz, 1H), 6.36 (d, *J* = 2.0 Hz, 1H), 6.16 (d, *J* = 2.0 Hz, 1H), 5.42 (d, *J* = 7.6 Hz, 1H), 3.52 (dd, *J* = 11.4 Hz, 1H), 3.37 (d, *J* = 10.8 Hz, 1H), 3.29 (m, 1H), 3.27 (m, 1H), 3.26–3.04 (m, 1H). **^13^C-NMR** (100 MHz, DMSO-*D*_6_) δ (ppm): 179.5 (C-4), 166.0 (C-5), 163.0 (C-7), 159.0 (C-2), 158.4 (C-9), 149.8 (C-4′), 145.9 (C-3′), 135.6 (C-3), 123.2 (C-1′), 123.1 (C-6′), 117.6 (C-5′), 115.9 (C-2′), 105.7 (C-10), 104.4 (Glc-C-1″), 99.9 (C-6), 94.7 (C-8), 78.4 (Glc-C-4″), 78.1 (Glc-C-2″), 75.7 (Glc-C-3″), 71.2 (Glc-C-5″), 62.5 (Glc-C-6″). These data are in agreement with those reported by Park et al. [24]. The spectra are shown in the Supplementary Materials (Figure S1 and Figure S2).

The identification of Isoquercitin (syn. isoquercitrin), a well-known flavonoid with antioxidant, anti-inflammatory, and cardioprotective properties, highlights AT’s medicinal potential. Isoquercitrin has previously been studied for its role in cardiovascular health, which aligns with AT’s traditional uses as a cardiotonic plant [25].

### 2.3. The Bioactivity Studies

#### 2.3.1. Cell Viability Assay

**Figure 2:**
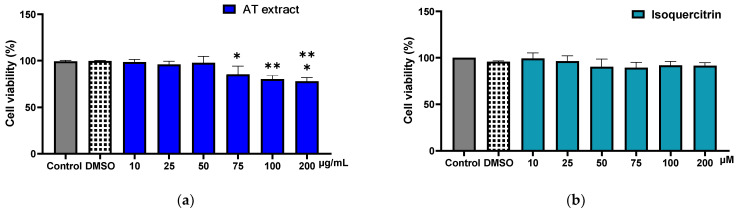
Effects of AT extract (**a**) and isoquercitrin (**b**) on the viability of RAW 264.7 cells. (**a**) Cells were cultured with AT extract at concentrations of 10, 25, 50, 75, 100, and 200 μg/mL. (**b**) Cells were cultured with isoquercitrin at concentrations of 10, 25, 50, 75, 100, and 200 μM. Cell viability was measured using the CCK-8 assay after 24 h. Data represent the mean ± SD of at least three independent experiments. * *p* < 0.05, ** *p* < 0.01, *** *p* < 0.001 (vs. control).

The AT extract demonstrated no cytotoxic effects on murine macrophages at concentrations up to 50 µg/mL, as determined by the CCK-8 assay. Cell viability remained consistent with that of the control group (Figure 2). Consequently, further analyses were performed using concentrations of 50 µg/mL or below. Additionally, isoquercitrin exhibited no toxicity at any of the tested concentrations.

#### 2.3.2. Determination of NO Production

**Figure 3:**
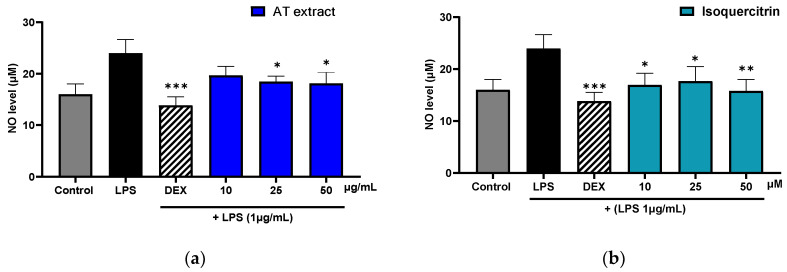
Effects of AT extract (**a**) and isoquercitrin (**b**) on NO production in RAW 264.7 cells. (**a**) Cells were treated with LPS (1 µg/mL), and either DEX (5 µM) or AT extracts at concentrations of 10, 25, and 50 µg/mL (**b**) Cells were treated with LPS (1 µg/mL) and either DEX (5 µM) or isoquercitrin at concentrations of 10, 25, and 50 µM. After 24 h, NO production was assessed using the Griess assay. Data are presented as the mean ± SD from at least three independent experiments. * *p* < 0.05, ** *p* < 0.01, *** *p* < 0.001 (compared to LPS-treated cells).

The results in Figure 3 demonstrate the significant impact of the AT extract and isoquercitrin on NO production in LPS-stimulated RAW 264.7 macrophages. NO production was measured based on the amount of nitrite in the media, and the reductions were calculated relative to the LPS-treated group. Treatment with LPS alone caused a marked increase in NO production (23.99 µM) compared to the control group (15.99 µM), indicating an inflammatory response. This increase was effectively suppressed by dexamethasone (5 µM), which reduced NO production by 42.37% (13.83 µM) compared to the LPS-only group.

The AT extract exhibited dose-dependent inhibition of NO production. At the highest concentration tested (50 µg/mL), NO production was reduced by 24.54% (18.11 µM) compared to the LPS-only group. At lower concentrations, 10 µg/mL and 25 µg/mL, the extract reduced NO production by 18.04% (19.66 µM) and 19.58% (19.29 µM), respectively.

Isoquercitrin also displayed dose-dependent effects, with the highest concentration (50 µM) reducing NO levels by 34.26% (15.77 µM), approaching the suppression observed with dexamethasone. Intermediate concentrations of isoquercitrin (10 µM and 25 µM) resulted in reductions of 29.31% (16.96 µM) and 26.37% (17.66 µM), respectively.

#### 2.3.3. Determination of Cytokine Production in Cell Supernatant

**Figure 4:**
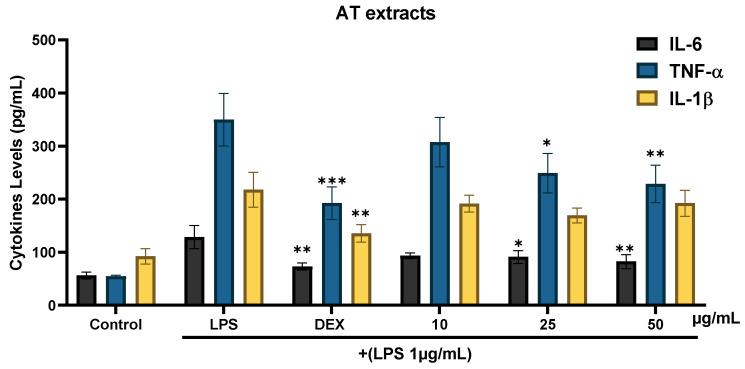
Effects of AT extract on cytokine production in RAW 264.7 cells. Cells were treated with LPS (1 µg/mL) and either DEX (5 µM) or AT extract at concentrations of 10, 25, and 50 µg/mL. After 24 h, cytokine production was assessed using ELISA. Data are presented as the mean ± SD from at least three independent experiments. * *p* < 0.05, ** *p* < 0.01, *** *p* < 0.001 (compared to LPS-treated cells).

**Figure 5:**
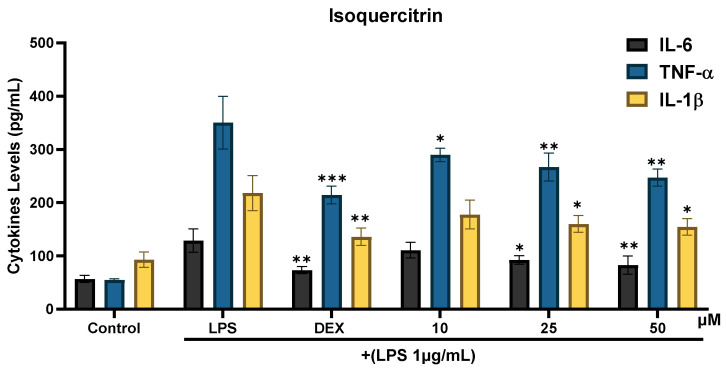
Effects of isoquercitrin on cytokine production in RAW 264.7 cells. Cells were treated with LPS (1 µg/mL) and either DEX (5 µM) or isoquercitrin at concentrations of 10, 25, and 50 µM. After 24 h, cytokine production was assessed using ELISA. Data are presented as the mean ± SD from at least three independent experiments. * *p* < 0.05, ** *p* < 0.01, *** *p* < 0.001 (compared to LPS-treated cells).

RAW 264.7 cells were exposed to the extracts along with LPS to determine the effect of AT extract on pro-inflammatory cytokines. Both AT extract (Figure 4) and isoquercitrin (Figure 5) demonstrated significant anti-inflammatory activity by reducing IL-6, TNF-α, and IL-1β levels.

The AT extract exhibited a dose-dependent reduction in cytokine levels. At the highest concentration tested (50 µg/mL), it inhibited IL-6 by approximately 63.6% and TNF-α by 34.6%, compared to the LPS-only group. However, its effect on IL-1β was less pronounced, with a maximum reduction of 21.2% at 50 µg/mL. In comparison, dexamethasone (5 µM) consistently demonstrated more significant inhibition across all three cytokines, reducing IL-6 by 76.9%, TNF-α by 49.1%, and IL-1β by 57.9%.

Isoquercitrin also demonstrated significant anti-inflammatory activity. At its highest concentration (50 µM), isoquercitrin inhibited IL-6 by 49.2% and TNF-α by 38.2%, showing a stronger effect on TNF-α than the extract. Additionally, it exhibited a pronounced inhibitory effect on IL-1β, reducing levels by 52.7%, which was comparable to that of dexamethasone (58.9%). These findings highlight the broad anti-inflammatory activity of isoquercitrin, particularly against IL-1β.

## 3. Discussion

The fingerprinting study of AT water and ethanol extracts showed the presence of various polyphenols in the samples. In addition to the traces of cardiac glycosides (strophanthidin and cymarin) and an alkaloid (embelin) in the crude extracts, the AT extract was predominantly rich in polyphenols. It contained flavonoids (e.g., adonitol, kaempferol derivatives), coumarins (e.g., methylumbelliferyl glucuronide), organic acids (e.g., malic acid), and organic alcohol (e.g., adonitol). Flavonoids were the most represented group of secondary metabolites present in the analyzed samples, with the highest peaks in the recorded chromatogram (see Figure S1 in the Supplementary File). As shown in Figure S1, cardiac glycosides and alkaloids were localized after the 20th minute of analysis, while phenolic compounds and other substances eluted from the column earlier. For example, malic and citric acids were detected in the first few minutes of the analysis, and fatty acids were detected at the end of the recorded chromatograms. Flavonoids and coumarins were detected between the 13th and 20th minute.

Cardiac glycosides are marker compounds of plants of the genus *Adonis* and are responsible for their cardiotonic actions. In the French Pharmacopoeia, the *Adonis vernalis* plant is standardized by the content of cymarin, where the sum of cardiac glycosides in terms of cymarin should be in the range from 0.01% *w*/*w* to 0.03% *w*/*w* [26]. According to the German Homeopathic Pharmacopoeia requirements, the content of the sum of cardiac glycosides in terms of cymarin is regulated within the range of not less than 0.0005 and not more than 0.0050% [27]. The results of this study show that the content of cardiac glycosides in the investigated extracts was low. Both compounds (strophanthidin and cymarin), which were only traced during this study, have cardiotonic effects and are used clinically for treating heart failure. Moreover, it has been proven that strophanthidin has an antitumor effect on human lung adenocarcinoma A549 cells [28]. The previously published data have also shown that cymarin inhibits MCF-7 cell proliferation by affecting PAX6 expression, making it a promising candidate for breast cancer treatment [29].

It is important to emphasize that the presence of cardiac glycosides in the investigated extracts was scarce. The relevant *m*/*z* values for the two tentatively identified compounds were below the detection limits, and we could not obtain MS/MS spectra for them. This may be due to the applied extraction protocol involving the use of solvents with high polarity. The results of the present study show that solvents with high polarity efficiently extract flavonoids and other polar secondary metabolites. Previously, it was demonstrated that the use of a 70% water-ethanol solution provided the maximum yields of flavonoids (1.22 ± 0.07%) and phenolic compounds (3.92 ± 0.17%) [30], which is in line with the current findings. Under these extraction conditions by other authors, the content of cardiac glycosides was 0.72 ± 0.07%. In previous studies, less-polar solvents, such as chloroform or acetone, were found to be more effective for extracting cardiac glycosides in a mixture with ethanol. Other authors have used chloroform-alcohol mixtures at 2:1 and 3:2 ratios to obtain higher glycoside yields [31]. These data are essential as they let us state that polyphenols are the major active ingredients of AT extracted using polar solvent mixtures and promise a variety of pharmacological properties that are beneficial to the human body.

Among the tentatively identified constituents of the extracts, embelin was identified. This benzoquinone alkaloid was previously found in the red berries of *Embelia ribes* plants. Several results of in vivo studies have proven that embelin has cardioprotective effects and reduces myocardial damage by enhancing antioxidant defense in isoproterenol-induced myocardial infarction in rats [32,33], significantly lowering serum levels of cardiac enzymes such as CK-MB, LDH, and AST in rats with isoproterenol-induced myocardial infarction, blocking myocardial apoptosis and necrosis, and improving cardiac function and hemodynamics after resuscitation [34,35]. In addition, in vivo and in vitro methods have proven the antitumor effect of embelin in various cancers, including breast cancer [36,37], prostate cancer [38,39,40], liver cancer [41,42], pancreatic cancer [43,44,45], colorectal cancer [46,47], gastric cancer [48], leukemia [49,50], and multiple myeloma [51], where it can effectively inhibit tumor cell proliferation and migration, induce tumor cell apoptosis, and inhibit tumor invasion, metastasis, and angiogenesis.

The results of chromatographic analysis show that the largest proportion of compounds belong to the flavonoid group. Flavonoids accounted for 48.93% of all identified compounds, and kaempferol derivatives accounted for 38.77%. Adonivernith (luteolin-8-hexityl monoxyloside), a compound named for its first detection in *Adonis vernalis,* is characteristic of many species of the genus [52]. Adonivernitol is present in large amounts in most organs of plants of the genus *Adonis*, especially in sepals, where it probably gives them a bright yellow color [53]. The results of the authors’ study showed that its concentration was exceptionally high in parasite-infected plants. Thus, this flavonoid in *Adonis* flowers performs both attractive and protective functions. However, the pharmacological activity of adonivernith is yet to be studied and requires further investigation.

Kaempferol and its derivatives, which are widely found in medicinal plants, are among the most active and important natural anti-inflammatory compounds [54]. Traces of aglycons, like kaempferol and higher quantities of glycoside derivatives of kaempferol, were detected in the tested extracts. Among them, kaempferol acetylglucoside-glucoside isomers, kaempferol glucosyl-glucoside, kaempferol sinapoylglucosyl-galactoside, a diglycoside derivative of kaempferol, and kaempferol acetylgalactoside were assigned (Table 1). Kaempferol has been proven in several scientific studies to have a positive effect on reducing the risk of chronic diseases such as cancer, liver disease, obesity, and diabetes. It has anti-inflammatory properties and is used to treat various acute and chronic inflammation-related diseases, including intervertebral disc degeneration, colitis, postmenopausal bone mass loss, and acute lung injury. Recent data on kaempferol’s biological and pharmacological activities indicate its marked antioxidant activity against red blood cells and its ability to inhibit the growth of bladder cancer cells by inducing apoptosis and blocking the S-phase of the cell cycle [55]. The proven biological activity of kaempferol and its derivatives underlines the importance of *A. tianshanicus* as a source of pharmacologically valuable metabolites.

Additionally, sinapoylsaponarin, which was annotated in this study in the tested samples, showed potential radical-scavenging activity in previous studies. These data indicate the possibility of its use as an antioxidant agent capable of neutralizing free radicals [56].

Scientific studies have provided evidence of the presence of different groups of metabolites in the extracts of *Adonis* spp. Adoniverinit was isolated from *A. vernalis* and *A. leiosepala*. The flavonoid orientin has been identified in *A. vernalis*, *A. coerulea*, *A. amurensis*, *A. appenina*, *A. wolgensis*, *A. tianschanica*, and *A. turkestanica*; luteolin—in *A. vernalis*, *A. coerulea*, *A. amurensis*, *A. mongolica*; apigenin—in *A. coerulea* and *A. amurensis*. In addition, in *A. vernalis*, homoaduniversitin, homoorientin, isoorientin, and vitexin were found; in *A. coerulea*—isoorientin and 7-glucoside of luteolin; in *A. mongolica*—kaempferin, kaempferol, 7-glucoside of luteolin, and *β*-glucoside of orientin; in *A. amurensis*—apigenin-7-*O-β-D*-glucuronide and isoquercitrin; and in AT and *A. turkestanica*—adonivernitol [57,58].

It is known that annual species of the genus *Adonis*, *A. annua*, and *A. aestivalis*, which have red flowers, contain the carotenoid astaxanthin [59,60]. At the same time, the carotenoid composition of *A. annua* petals, includes hydroxyecchinenone, adonirubin and adonixanthin, adonixanthin diester, 3-hydroxy-echinenone ester, cis-astaxanthin diester, trans-astaxanthin diester, adonirubin ester, cis-astaxanthin complex monoester and trans-astaxanthin complex monoester. Astaxanthin was found in the perennial species of *A. amurensis*, as were the fatty acid ester components of the ketocarotenoids astaxanthin, 3-hydroxyecchinenone, 3,3′-dihydroxyecchinenone, and 3-hydroxycanthaxanthin, as well as 3,4-diketo-*β*-carotene and 3,4,4′-triketo-*β*-carotene [61].

Also previously, coumarins—umbelliferone and scopoletin—were isolated from the roots of *A. amurensis*, *A. wolgensis*, *A. leiosepala*, and *A. mongolica* [62,63,64,65].

The results of studying leaves and stems of *A. wolgensis* for fatty acid content showed the highest prevalence of linolenic (45.83%) and oleic (47.54%) acids in the plants [66].

Among other constituents of *Adonis* spp., stigmast-4-ene-3,6-dione, stigmast-4-ene-3-one *6β*-hydroxy, *β-D*-glucopyranoside, palmitic acid, adonite, β-sitosterol, 1-hentriacontanol, and *p*-formylcinnamic acid were found in *A. coerulea* [67], a new tetraoside, sugoroside, was identified in *A. chrysocyathus* extracts, and a five-atom alcohol, adenitol, was identified in *A. mongolica* and *A. leiosepala*. In addition, adoligosides A-E were isolated from *A. aleppica*, and three lignans, namely, pinoresinol, pinoresinol-8-*O-β-D*-glucopyranoside, and decarboxy-rosmarinic acid-4-*O*-(1 → 4)-galactosylramnoside, were isolated from *A. amurensis* [68].

The multitude of metabolites identified in *Adonis* species warrants further study and isolation trials, as many of them exhibit important pharmacological potential and can be used as isolated single molecules to treat various disorders. An example is the isolation of isoquercitrin. This study introduced an interesting, repetitive, cheap, and easily up-scalable method for recovering this flavonoid from the extracts of AT using a liquid-liquid chromatography technique, namely HSCCC.

Isoquercitrin, the primary compound isolated from AT, demonstrated significant anti-inflammatory effects in this study, particularly through the reduction of NO production and the suppression of pro-inflammatory cytokines IL-6, TNF-α, and IL-1β. These results align with the findings of Lee et al. [69], who showed that isoquercitrin inhibits the NF-κB signaling pathway by preventing the degradation of IκBα, a key inhibitor of NF-κB. By blocking NF-κB activation, isoquercitrin downregulates the expression of pro-inflammatory mediators, such as iNOS and COX-2, leading to reduced production of NO and prostaglandins like PGE2. This mechanism likely highlights the strong anti-inflammatory response observed in our study.

Additionally, the ability of isoquercitrin to modulate MCP-1 expression, as demonstrated in previous studies [70], suggests a broader anti-inflammatory role by regulating monocyte recruitment and macrophage differentiation. Although MCP-1 was not evaluated in this study, the suppression of IL-1β, TNF-α, and IL-6 expression supports this regulatory mechanism. The capacity of isoquercitrin to inhibit COX-2 and iNOS further enhances its therapeutic potential, particularly under inflammatory conditions.

The AT extract exhibited moderate anti-inflammatory effects, reducing IL-6 and TNF-α levels, although its impact on IL-1β was less pronounced. The broader mix of polyphenols and flavonoids present in the extract likely contributes to its activity, although it is less potent than isoquercitrin alone. This is consistent with the idea that polyphenol-rich extracts, although less concentrated in any single compound, offer cumulative anti-inflammatory benefits by modulating several pathways.

AT extract also demonstrated anti-inflammatory effects, although less pronounced than those of isoquercitrin alone. It reduced IL-6, TNF-α, and to a lesser extent IL-1β, indicating that the extract’s broad mixture of polyphenols and flavonoids may act synergistically to provide moderate anti-inflammatory effects.

Taken together, these results suggest that AT extract contains negligible quantities of cardiac glycosides and enough quantities of phenolics, represented by its main isolated compound, isoquercitrin. This warrants the iinvestigation of advanced pharmaceutical formulations for this extract, aiming to produce a mild natural anti-inflammatory medicine.

## 4. Materials and Methods

### 4.1. Materials

Dimethyl Sulfoxide-*D*_6_ (DMSO, 99.9% purity) (D2650) was obtained from Sigma-Aldrich (Arklow, Ireland). Lipopolysaccharide (LPS) from Escherichia coli 0111:B4 (L4391), fetal bovine serum (FBS, F7524), and Dulbecco’s Modified Eagle’s Medium (DMEM, D0822) were also purchased from Sigma-Aldrich (Arklow, Ireland). The Cell Counting Kit-8 (CCK-8) was sourced from Dojindo (Rockville, MD, USA), while dexamethasone was acquired from Sigma-Aldrich (D4902). Griess reagent (G4410) and sodium nitrite (237213) were obtained from Sigma-Aldrich. Mouse ELISA kits, including all test reagents for IL-6, IL-1β, and TNF-α cytokine detection, and ELISA plates (Nunc™ MaxiSorp™ ELISA Plates) were purchased from BioLegend (San Diego, CA, USA).

### 4.2. The Plant Material

The object of the study—AT (Adolf.) Lipsch. was collected during the flowering period in May 2022 in Saty village, Kegen district, Almaty region, Republic of Kazakhstan. The geographic coordinates of the collection site are 43.0708, 78.4099.

The collected raw material was identified by the “Institute of Botany and Phytointroduction” of the Committee of Forestry and Wildlife of the Ministry of Ecology, Geology and Natural Resources of the Republic of Kazakhstan, and certificates of identification were obtained.

### 4.3. Extraction of Plant Material

The extraction was performed in 50 mL falcons using 5 g of powdered plant material and 50 mL of solvent. The extracts were prepared by ultrasound extraction for 30 min at room temperature. After extraction, the falcons were centrifuged at 1500 rpm, and the supernatants were collected, filtered through a filter paper, and evaporated to dryness at 45 °C. Three types of extracts were prepared using the same methodology, with ethanol 99% water and 50% ethanol: water mixture as extracting solvents, to obtain a wider range of polarities that would favor more efficient extraction. The dried residues were stored in the fridge for no longer than two weeks until analysis. For the HPLC-MS analyses, the dried residues were re-dissolved in the original solvents to give a concentration of 10 mg/mL.

For the bioactivity studies, 1 g of AT ethanol: water (50:50 *v*/*v*) extract was dissolved in 100% DMSO at a concentration of 100 mg/mL as the stock of the extracts.

### 4.4. NMR Identification

Structural elucidation of the isolated compound was performed using a Bruker Avance 400 instrument (Bremen, Germany) operating at 400 MHz for proton (^1^H NMR) and 100 MHz for carbon (^13^C NMR) magnetic resonance. The sample was dissolved in deuterated dimethyl sulfoxide (DMSO-*D*_6_). Spectral analysis was carried out using the Mestrenova software (version 14.3.3-33362).

### 4.5. HPLC-MS-ESI-QTOF-MS/MS Fingerprinting of the Extract

The qualitative analysis of the tested samples was performed using an analytical platform—HPLC-ESI-QTOF-MS/MS from Agilent Technologies (Santa Clara, CA, USA)—composed of a high-performance liquid chromatograph (1200 Series) equipped with a binary pump, an autosampler, a degasser, a UV detector, and a thermostat that was coupled with a mass detector (G6530B) with the jet stream ionization source and a low flow rate pump for the calibration mixture injection. The following chromatographic conditions were used for the analysis of the tested samples: temperature of 20 °C, flow rate of 0.2 mL/min, injection volume of 10 µL, and gradient of acetonitrile with 0.1% formic acid (solvent B) in 0.1% aqueous solution of formic acid according to this program: 45 min—60% of B, 46 min—95% of B, 55 min—95% of B. The chromatographic separation of metabolites was performed on an RP-18 Zorbax Eclipse Plus column (150 mm × 2.1 mm, pore size of 3.5 µm). The mass spectrometer was operated in both positive and negative ion modes, with 3000 V capillary voltage, 1000 V nozzle voltage, 110 V fragmentor voltage, 65 V skimmer voltage, 275 and 325 °C gas and sheath gas temperatures, respectively, with 12 L/min gas flow setting and 35 psig nebulizer pressure. Collision energies of 10 V and 20 V were selected.

All runs were performed using a calibrated instrument, and the calibration mixture was dosed with the sample. The MS/MS spectra of a given molecular feature were collected twice, and then the given *m*/*z* was excluded for the following 0.3 min. The data were handled by the Mass Hunter Workstation (version B.10.00, Agilent Technologies, Santa Clara, CA, USA). The positive ion mass chromatograms were found to be richer in metabolites, and they were later analyzed in Mass Hunter Profinder 10.0, and Mass Profiler Professional (v.15.1 Agilent Technologies, Santa Clara, CA, USA) programs to determine the molecular features that were differentiating the extracts. For this purpose, the extraction of molecular features was performed in the cef format with a mass tolerance of 10 ppm and an abundance of more than 1% in the total extract. PCA analysis was performed in the Mass Profiler Professional Program using 376 molecular features in a one-way ANOVA with asymptotic *p*-value computation and Benjamini-Hochberg multiple testing correction (*p* < 0.05, fold change of 2).

The identification of metabolites was based on accurate mass measurements, MS/MS fragmentation patterns, and consideration of the scientific literature and open databases (Metlin MS collection of metabolites and small molecules).

### 4.6. Fractionation of the Extract

High-Speed Counter-Current Chromatography (HSCCC) was performed using an IntroPrep™-Quattro system (Malvern, PA, USA) with a 136 mL PTFE coil and a 6 mL sample loop. A solvent system consisting of ethyl acetate, n-butanol, and water (2:3:5) was used with the aqueous phase serving as the stationary phase. The separation was carried out in the “head-to-tail” elution mode, where the upper organic layer acted as the stationary phase and the aqueous layer as the mobile phase. After equilibrium was reached, the system was rotated at 850 rpm and 4 mL fractions were collected every 2 min, resulting in 80 fractions per run.

Thin Layer Chromatography (TLC) was used to analyze the fractions, employing a solvent mixture of ethyl acetate, formic acid, and acetic acid (5:1.3:1.3), with visualization by NP-PEG reagent. Fractions with similar profiles were combined and further purified using Sephadex LH-20 (Sigma Aldrich, Arklow, Ireland) with methanol as the eluent.

### 4.7. Bioactivity Studies

#### 4.7.1. Cell Culture

The RAW264.7 cell line used in this study was kindly provided by Dr. Maria Santos-Martinez, Associate Professor of Pharmacy at Trinity College Dublin. Murine macrophages (RAW264.7 cells) were grown in Dulbecco’s Modified Eagle’s Medium (DMEM)/high glucose supplemented with 10% fetal bovine serum (FBS). The cells were incubated at 37 °C in a humidified atmosphere containing 5% CO_2_.

#### 4.7.2. Cell Viability Assay

Once RAW264.7 cells reached 80% confluency, they were detached and seeded in 96-well plates (1 × 10^5^ cells/well). After 24 h, the cells were treated with AT extract at 10, 25, 50, 75, 100, or 200 μg/mL or isoquercitrin at 10, 25, 50, 75, 100, or 200 μM in serum-free media. Cell Counting Kit-8 (CCK-8) assay was used to quantitatively assess RAW264.7 cells viability after exposure to the extracts following the manufacturer’s instructions. Briefly, following incubation with various concentrations of the test drugs or the vehicle diluted in serum-free culture medium for 24 h, the medium was removed, and cells were incubated for another 2 h in the presence of the CCK-8 reagent. Subsequently, a microplate reader (BioTek Instruments, Inc., Winooski, VT, USA) was used to measure the absorbance (OD) at 450 nm. Relative cell viability was calculated using the following equation:

(1) $$Relative\;cell\;viability\;=\;\frac{OD\;of\;experimental\;group}{OD\;of\;control\;group}*100$$

#### 4.7.3. Data Analysis

All experiments were repeated three times. Results are expressed as mean ± standard deviation (SD). Differences between the sample and LPS groups were determined using one-way ANOVA and Dunnett/Duncan’s multiple post-test when appropriate. *p*-values less than 0.05 (*p* < 0.05) were used as the significant level.

#### 4.7.4. Determination of NO Production

RAW 264.7 cells were seeded in 96-well plates at a density of 1 × 10⁵ cells per well and cultured in DMEM supplemented with 10% FBS at 37 °C in a humidified atmosphere of 5% CO_2_ for 24 h. After reaching confluency, the cells were treated with LPS (1 µg/mL) to induce NO production, followed by dexamethasone (DEX, 5 µM) as a positive control or AT extract at concentrations of 10, 25, and 50 µg/mL, or Isoquercetrin at concentrations of 10, 25, and 50 µM.

After 24 h of incubation, the cell culture supernatants were collected and stored on ice. Equal volumes of the collected supernatants and pre-prepared 1× Griess reagent (prepared by dissolving the supplied powder in 250 mL of nitrite-free water and mixing for five minutes) were combined in a 96-well plate. The samples were incubated at room temperature for 15 min to allow color development. Absorbance was measured at 540 nm using a microplate reader (BioTek Instruments, Inc., USA).

A standard curve was prepared using sodium nitrite dissolved in nitrite-free water, and the concentrations of NO in the samples were calculated using this curve.

#### 4.7.5. Determination of Cytokine Production in Cell Supernatant

After treating the cells with LPS (1 µg/mL), DEX (5 µM), AT extract (10, 25, and 50 µg/mL), or isoquercitrin (10, 25, and 50 µM) for 24 h, the RAW 264.7 cell culture supernatants were harvested. The concentrations of IL-6, IL-1β, and TNF-α were measured using cytokine enzyme-linked immunosorbent assays (ELISA) following the manufacturer’s instructions.

Briefly, a 96-well ELISA plate was pre-coated with 100 µL of capture antibody diluted in Coating Buffer A incubated overnight at 4 °C. The plate was washed four times with Wash Buffer (PBS + 0.05% Tween-20) and blocked with 200 µL Assay Diluent A for 1 h at room temperature with shaking. After washing, 100 µL of either standard (ranging from 7.8 to 500 pg/mL) or cell culture supernatants (undiluted) were added to the wells and incubated for 2 h at room temperature with shaking.

The plate was washed again, and 100 µL of biotinylated detection antibody diluted in Assay Diluent A was added to each well, followed by 1-h incubation at room temperature with shaking. After washing, 100 µL of Avidin-HRP solution was added, and the plate was incubated for 30 min at room temperature. The wells were washed thoroughly, and 100 µL of TMB substrate solution (1:1 mixture of Substrate Solution A and Solution B) was added and incubated in the dark for 20 min. The reaction was stopped with 100 µL of Stop Solution, and the absorbance was read at 450 nm using a microplate reader.

Cytokine concentrations were determined by comparing the sample absorbance values to the standard curve generated from known concentrations of recombinant cytokines. All samples and standards were run in triplicate to ensure reproducibility.

## 5. Conclusions

*A. tianschanica*, an endemic species of Kazakhstan’s flora, represents a significant source of biologically active compounds, particularly flavonoids. The extensive analysis of the extracts from AT presented in this study demonstrated a marked anti-inflammatory effect. Notably, the capacity to cultivate AT opens new avenues for screening this endemic plant for a variety of biological properties, thereby facilitating its potential large-scale application in the pharmaceutical industry.

## Figures and Tables

**Table 1:** The tentatively identified metabolites of *Adonis tianschanica* in the HPLC-ESI-QTOF-MS/MS analysis (Diff—error of measurement, DBE—double bond and ring number, tr—traced with no MS/MS spectra registered).

No	Extract	Ion	Retention Time [min]	Compounds	Neutral Formula	Theoretical Mass	Precursor Ion *m*/*z*	Diff [ppm]	DBE	Products Ions [*m*/*z*]
	Cardiac glycosides									
1	50% EtOH	M+H^+^	19.54	Strophanthidin /adonitoxigenin	C_23_H_32_O_6_	405.2272	405.2294	−5.53	8	tr
2	50% EtOH	M+H^+^	20.96	Cymarin	C_30_H_44_O_4_	549.3058	549.3071	−2.35	-	tr
	Alkaloids									
3	50% EtOH	[M−H]^−^	22.65	Embelin	C_17_H_26_O_4_	293.1758	293.1782	−8.05	5	207.1737 96.9601
	Flavonoids									
4	50% EtOH	[M−H]^−^	17.95	Adonivernith (luteolin-8-hexityl monoxyloside)	C_26_H_28_O_15_	579.1355	579.1353	0.42	13	418.1571 285.0347
5	50% EtOH	[M−H]^−^	15.28/16.66	Kaempferol acetylglucoside-glucoside isomers	C_29_H_32_O_17_	651.1567	651.1548	2.87	14	532.12 430.0809 358.0664 328.0536 286.0481
6	50% EtOH	[M−H]^−^	14.32	Kaempferol glucosyl-glucoside	C_27_H_30_O_16_	609.1461	609.1454	1.08	13	490.1061 430.0855 358.0655 328.0539 310.0433
7	50% EtOH	[M−H]^−^	18.75	Kaempferol sinapoylglucosyl-galactoside	C_38_H_40_O_20_	815.2040	815.1944	7.74	19	785.1876 696.1604 610.1480 430.0847 357.0583 327.0518 309.0363 285
8	50% EtOH	[M−H]^−^	20.36	Kaempferol/luteolin	C_15_H_10_O_6_	285.0405	285.0409	−1.53	11	tr
9	50% EtOH	[M−H]^−^	20.52	A diglicoside derivative of kaempferol	C_32_H_30_O_15_	653.1512	653.1504	1.24	18	623.1344 448.0910 358.0660 327.0516
10	50% EtOH	[M−H]^−^	19.19/18.56	Sinapoylsaponarin	C_38_H_40_O_19_	799.2091	799.2079	6.13	19	757.1956 594.1546 414.0881 293.0445 223.0558
11	50% EtOH	[M−H]^−^	14.6	Isoorientin	C_21_H_20_O_11_	447.0933	447.0934	−0.03	12	369.0639 357.0599 327.0502 285.0390
12	50% EtOH	[M−H]^−^	15.39	A hexose-tetrose flavonoid derivative	C_27_H_30_O_15_	593.1512	593.1501	1.84	13	549.254. 414.0908 293.0458
13	EtOH	[M−H]^−^	15.7	Isovitexin	C_21_H_20_O_10_	431.0984	431.0989	−1.23	12	389.2163 311.0554 283.0605 251.0918
14	50% EtOH	[M−H]^−^	15.9	Orientin	C_21_H_20_O_11_	447.0933	447.0933	−0.03	12	394.0593 369.0585 357.0607 327.0497 285.0427
15	50% EtOH	[M−H]^−^	16.11	Orientin glucoside	C_27_H_30_O_16_	610.1532	609.1458	−0.3	13	490.1065 430.0866 358.0650 328.0540 310.0437
16	50% EtOH	[M−H]^−^	16.63	A hexose-tetrose flavonoid derivative	C_27_H_30_O_15_	593.1512	593.1501	1.84	13	474.1112 414.0888 294.0470
17	50% EtOH	[M−H]^−^	16.78	Vitexin	C_21_H_20_O_10_	431.0984	431.0988	−0.99	12	341.0672 311.0563 283.0593
18	50% EtOH	[M−H]^−^	17.39	Kaempferol acetylgalactoside	C_23_H_22_O_12_	489.1038	489.1029	2.75	13	357.0539 327.0502 297.0366
19	50% EtOH	[M−H]^−^	17.69	Isoquercitrin	C_21_H_20_O_12_	463.0882	463.0865	3.66	12	421.2052 301.0342 255.0351
20	50% EtOH	[M−H]^−^	21.19	Luteone glucoside	C_26_H_28_O_11_	515.1559	515.1538	4.04	13	414.0890 312.0576 283.0605
	Coumarins									
21	50% EtOH	[M−H]^−^	18.67	Methylumbelliferyl glucuronide	C_16_H_16_O_9_	351.0722	351.0704	4.99	9	205.0337 169.0123 163.0396 143.0337
	Organic acids									
22	50% EtOH	[M−H]^−^	2.01	Malic acid	C_4_H_6_O_5_	133.0142	133.0153	−7.86	2	115.0022
23	50% EtOH	[M−H]^−^	2.099	Citric acid	C_6_H_8_O_7_	191.0197	191.0198	−0.38	3	173.0096 111.0092 87.0098
	Fatty acids									
24	50% EtOH	[M−H]^−^	24.06	Hydroxypalmitic acid	C_16_H_32_O_3_	271.2279	271.2286	−2.69	1	253.2165 225.2221
25	50% EtOH	[M−H]^−^	24.21	Conjugated linoleic acid	C_18_H_32_O_2_	279.2330	279.2330	−0.16	3	261.2199 234.9184
26	50% EtOH	[M−H]^−^	26.89	Palmitic acid ethyl ester	C_18_H_36_O_2_	283.2643	283.2647	−1.22	1	-
	Organic alcohols									
27	50% EtOH	[M−H]^−^	1.93	Adonitol	C_5_H_12_O_5_	151.0612	151.0620	−5.28	0	119.0362 101.0251 89.0250

**Table 2:** The average relative percentage content calculated based on the peak area value (n = 3) of the respective metabolites in the analyzed extracts of *Adonis tianschanica* (The colors represent the percentage content value—red is the maximum measured peak area, whereas green pictures the minimum values).

Compound Name	50% EtOH	EtOH	H_2_O
Strophanthidin	-	-	-
/adonitoxigenin	-	-	-
Embelin	22.61	100	13.84
Adonivernith (luteolin-8-hexityl monoxyloside)	100	7.40	0.00
Kaempferol acetylglucoside-glucoside	100	72.02	1.46
Kaempferol 3-*O*-*β*-*D*-glucosyl-1->2bD-glucoside	65.91	100	0.00
Kaempferol sinapoylglucosyl-galactoside	12.44	100	0.32
Kaempferol/luteolin	8.18	100	0
A diglicoside derivative of kaempferol	6.69	100	0.00
Sinapoylsaponarin	12.96	100	0.29
Isoorientin	100	41.96	0
A hexose-tetrose flavonoid derivative	81.28	100	0.10
Isovitexin	100	77.48	0
Orientin	100	50.63	3.65
Orientin glucoside	58.80	100.00	2.37
A hexose-tetrose flavonoid derivative	94.64	100	2.80
Vitexin	100	51.49	5.53
Kaempferol acetylgalactoside	100	8.83	5.08
Isoquercitrin	100	77.33	0
Luteone glucoside	100	21.56	0
Methylumbelliferyl glucuronide	100	48.76	1.29
Hydroxypalmitic acid	7.30	100	1.09
Conjugated linoleic acid	0	100	0
Malic acid	100	52.31	0.45
Citric acid	100	8.19	2.06
Palmitic acid ethyl ester	12.25	100	0
Adonitol	35.97	100	10.60

## Data Availability

Data is contained within the article or Supplementary Materials.

## Supplementary Materials

The following supporting information can be downloaded at https://www.mdpi.com/article/10.3390/molecules29235754/s1, Figure S1. ^1^H-NMR spectrum of Isoquercitrin (400 MHz, DMSO-*D*_6_); Figure S2: ^13^C-NMR spectrum of Isoquercitrin (100 MHz, DMSO-*D*_6_); Table S1: The MS/MS spectra recorded for the majority of the tentatively identified molecules from Table 1.
