# Supplementary material for: Phytochemical Profile and Biological Activity of the Ethanol Extracts from the Aerial Parts of Adonis tianschanica (Adolf.) Lipsch. Growing in Kazakhstan
Source: Molecules. 2024 Dec 5;29(23):5754. doi: 10.3390/molecules29235754 (PMC11643382; doi:10.3390/molecules29235754)
Supplement: Supplementary file 1 [file molecules-29-05754-s001.zip › molecules-3339829-supplementary.pdf]

SUPPLEMENTARY FILE

Table S1. The MS/MS spectra recorded for the majority of the tentatively identified molecules from Table 1.

1.	Retention time [min]	Compounds	
2.	19.539	Strophanthidin/adonitoxigenin	traces
3.	20.958	Cymarin	traces
4.	22.652	Embelin	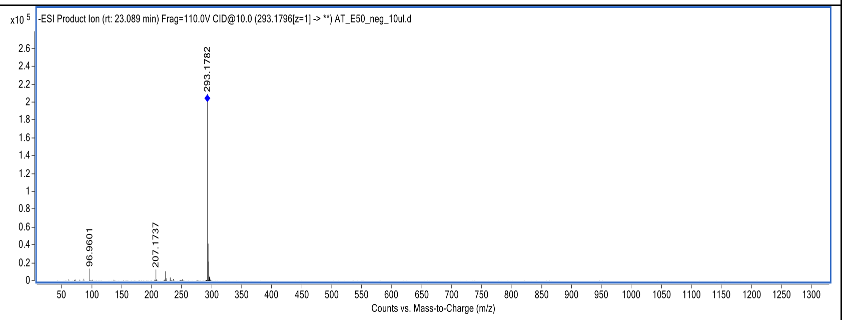
5.	17.95	Adonivernith (luteolin-8-hexityl monoxyloside)	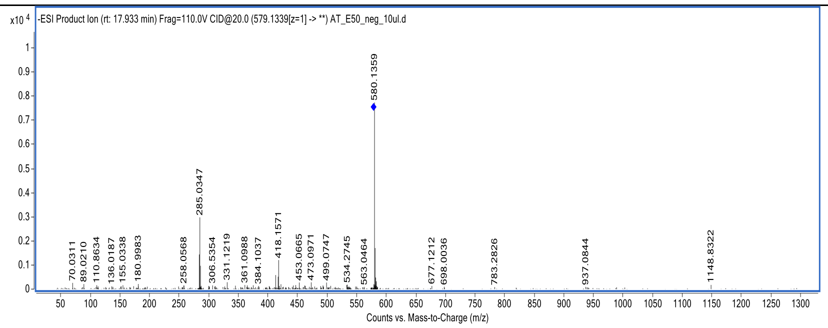
6.	15.279/ 16.659	Kaempferol acetylglucoside-glucoside	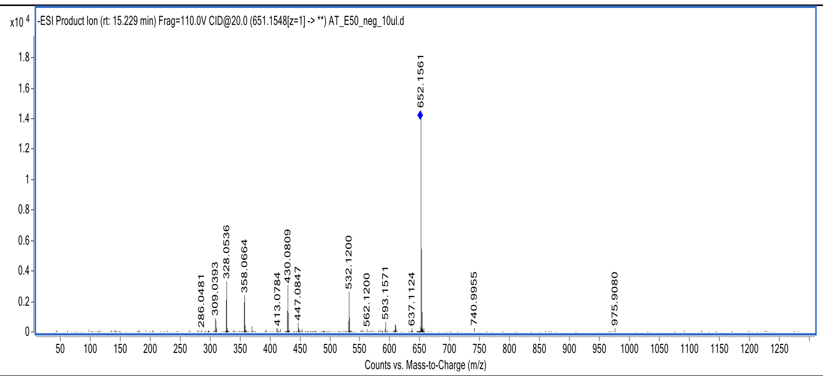
7.	14.318	Kaempferol 3-*O*-*β*-*D*-glucosyl-1->2bD-glucoside	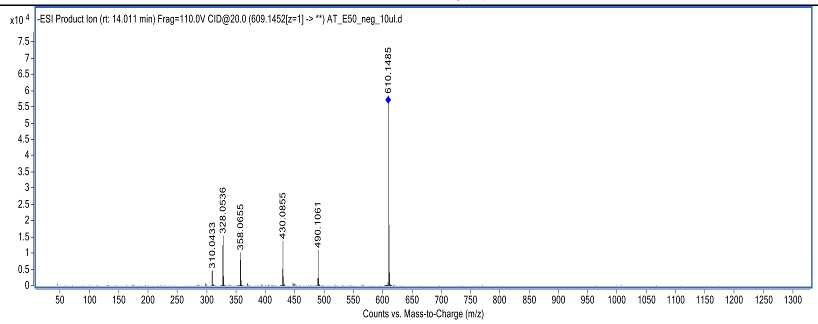

8.	18.75	Kaempferol sinapoylglucosyl-galactoside	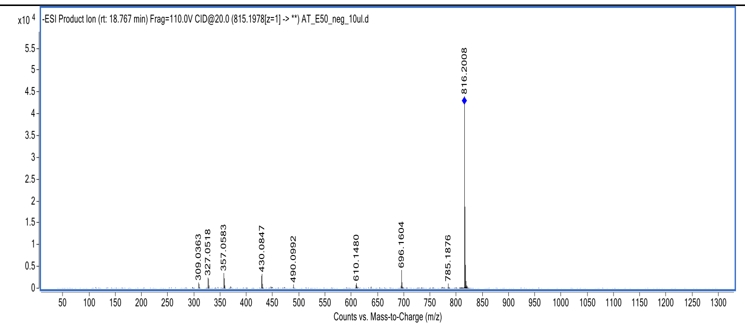
9.	20.36	Kaempferol /luteolin	traces
10.	20.517	A diglicoside derivative of kaempferol	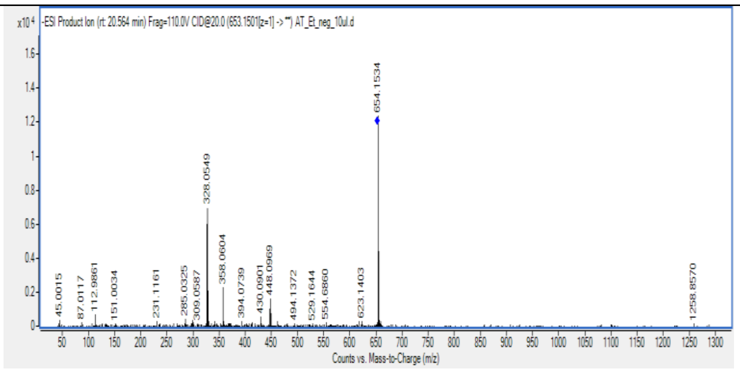
11.	19.19/18.56	Sinapoylsaponarin	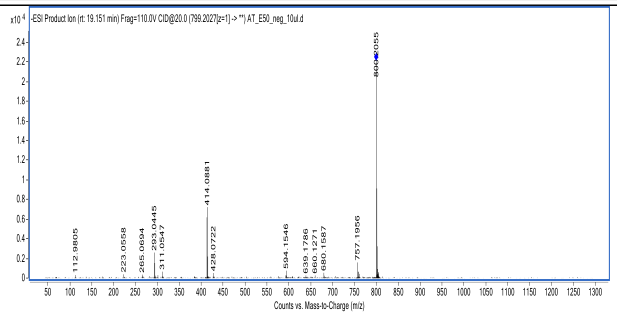
12.	14.6	Isoorientin	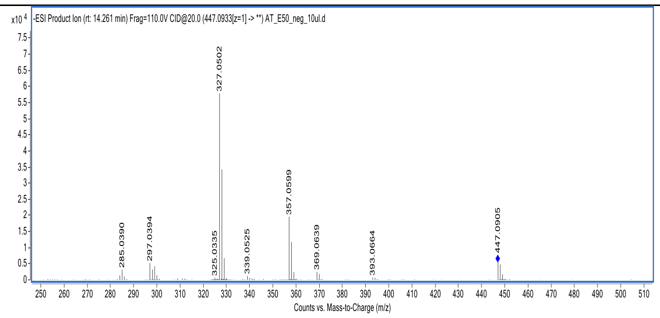
13.	15.39	A hexose-tetrose flavonoid derivative	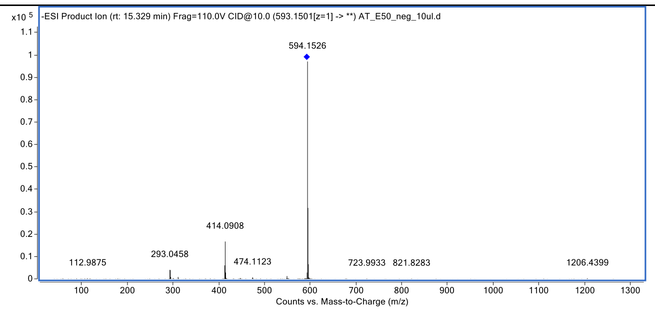

<table>
<tr><td>14.</td><td>15.7</td><td>Isovitexin</td><td>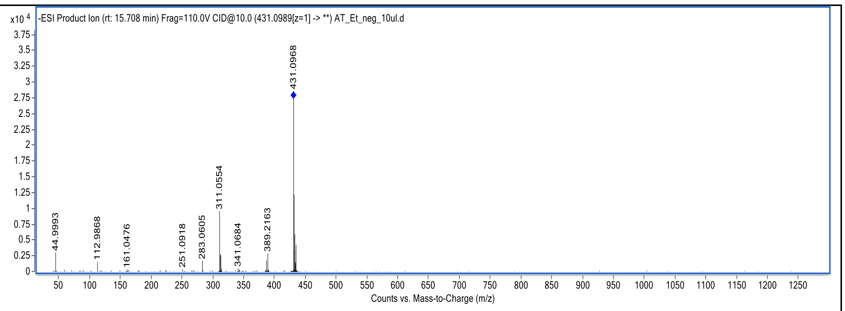
</td></tr>
<tr><td>15.</td><td>15.9</td><td>Orientin</td><td>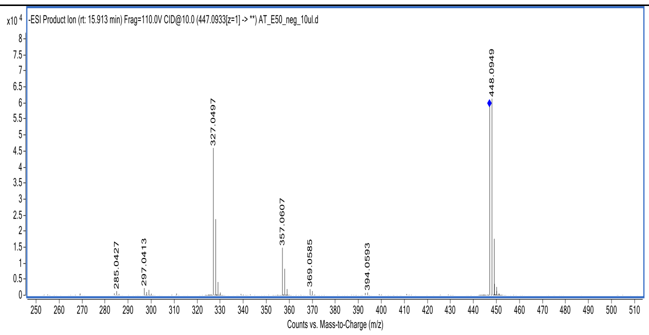
</td></tr>
<tr><td>16.</td><td>16.112</td><td>Orientin glucoside</td><td>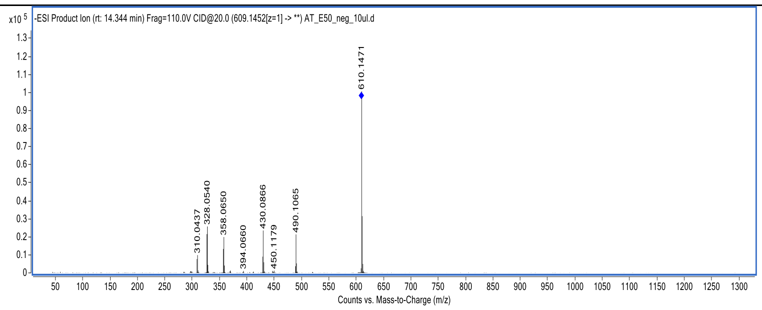
</td></tr>
<tr><td>17.</td><td>16.627</td><td>A hexose-tetrose flavonoid derivative</td><td>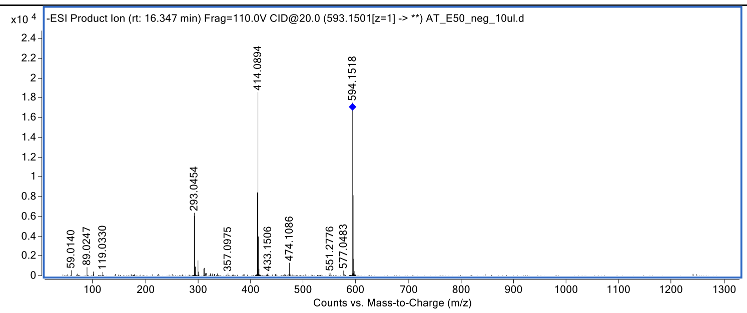
</td></tr>
<tr><td>18.</td><td>16.78</td><td>Vitexin</td><td>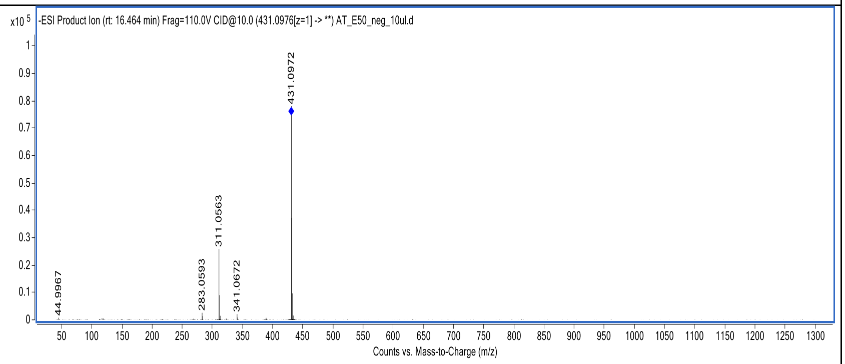
</td></tr>
</table>

19.	17.391	Kaempferol acetylgalactoside	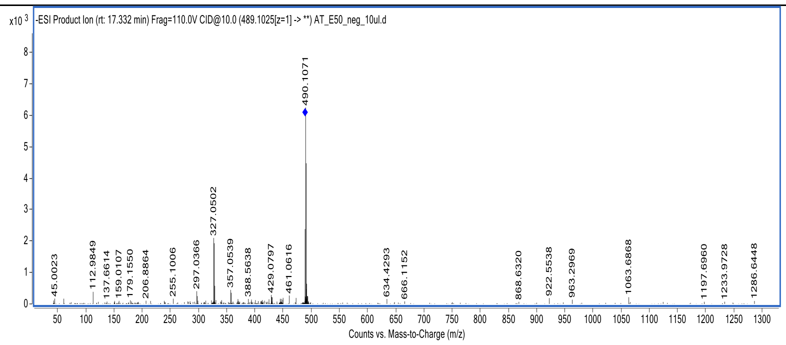
20.	17.692	Isoquercitrin	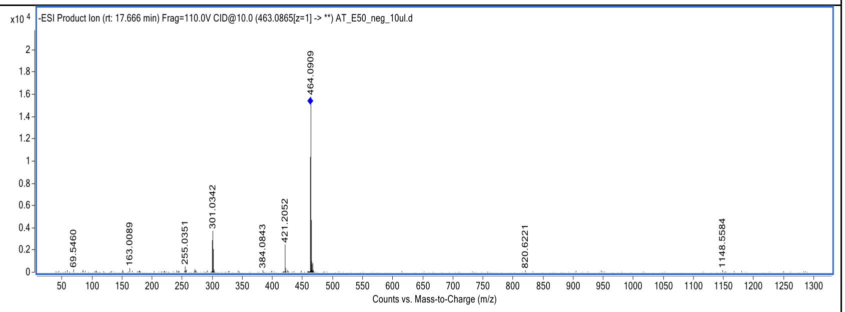
21.	21.19	Luteone glucoside	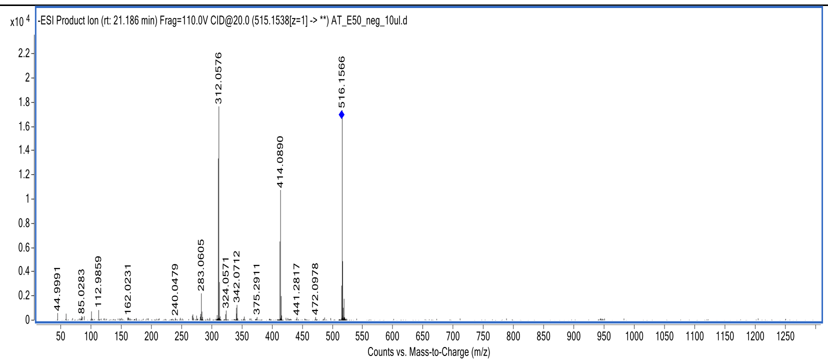
22.	18.67	methylumbelliferyl glucuronide	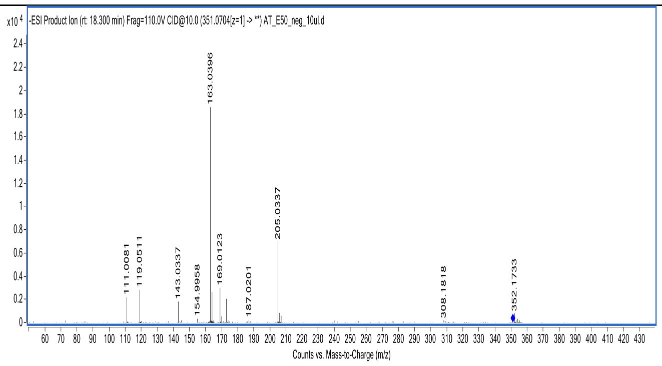
23.	24.058	Hydroxypalmitic acid	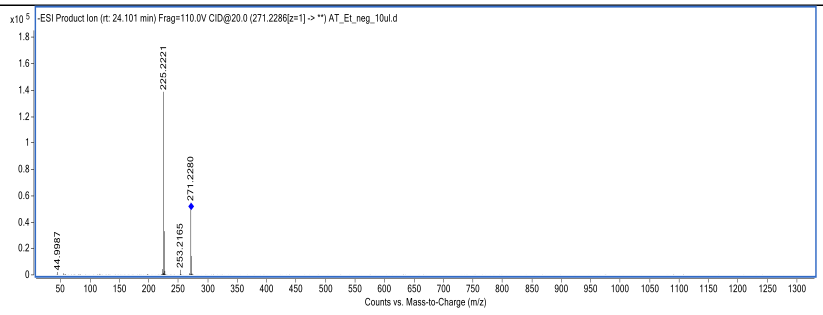

24.	24.207	Conjugated linoleic acid	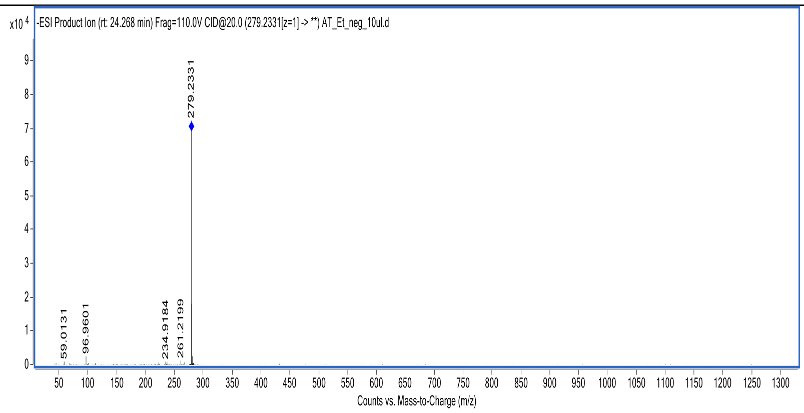
25.	2.01	Malic acid	
26.	2.099	Citric acid	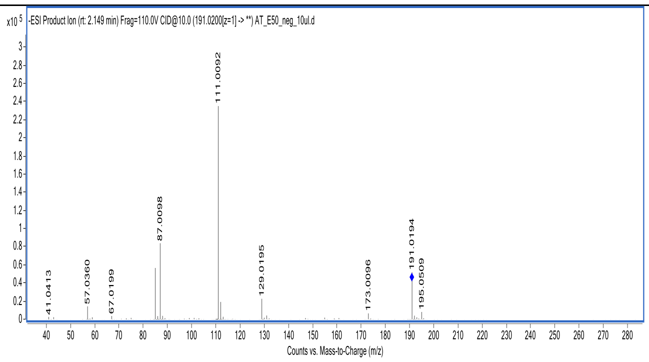
27.	26.886	Palmitic acid ethyl ester	
28.	1.932	Adonitol	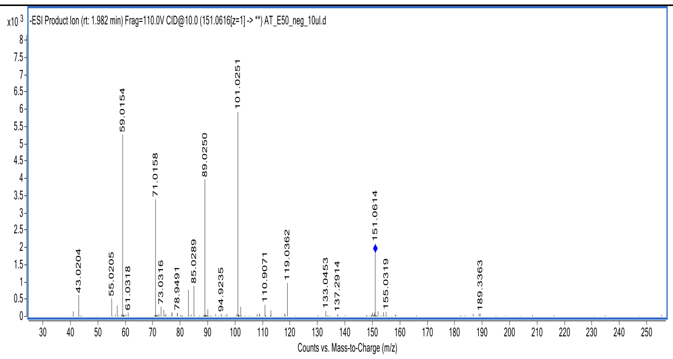

Isoquercetin:

^{1}H-NMR (400 MHz, DMSO-D_6)) δ (ppm): 7.55 (d, *J* = 2.2 Hz, 1H), 7.53 (dd, *J* = 8.5, 2.2 Hz, 1H), 6.80 (d, *J* = 8.5 Hz, 1H), 6.36 (d, *J* = 2.0 Hz, 1H), 6.16 (d, *J* = 2.0 Hz, 1H), 5.42 (d, *J* = 7.6 Hz, 1H), 3.52 (dd, *J* = 11.4 Hz, 1H), 3.37 (d, *J* = 10.8 Hz, 1H), 3.29 (m, 1H), 3.27 (m, 1H), 3.26–3.04 (m, 1H).

^{13}C-NMR (100 MHz, DMSO-D_6)) δ (ppm): 179.5 (C-4), 166.0 (C-5), 163.0 (C-7), 159.0 (C-2), 158.4 (C-9), 149.8 (C-4′), 145.9 (C-3′), 135.6 (C-3), 123.2 (C-1′), 123.1 (C-6′), 117.6 (C-5′), 115.9

(C-2′), 105.7 (C-10), 104.4 (Glc-C-1″), 99.9 (C-6), 94.7 (C-8), 78.4 (Glc-C-4″), 78.1 (Glc-C-2″), 75.7 (Glc-C-3″), 71.2 (Glc-C-5″), 62.5 (Glc-C-6″).

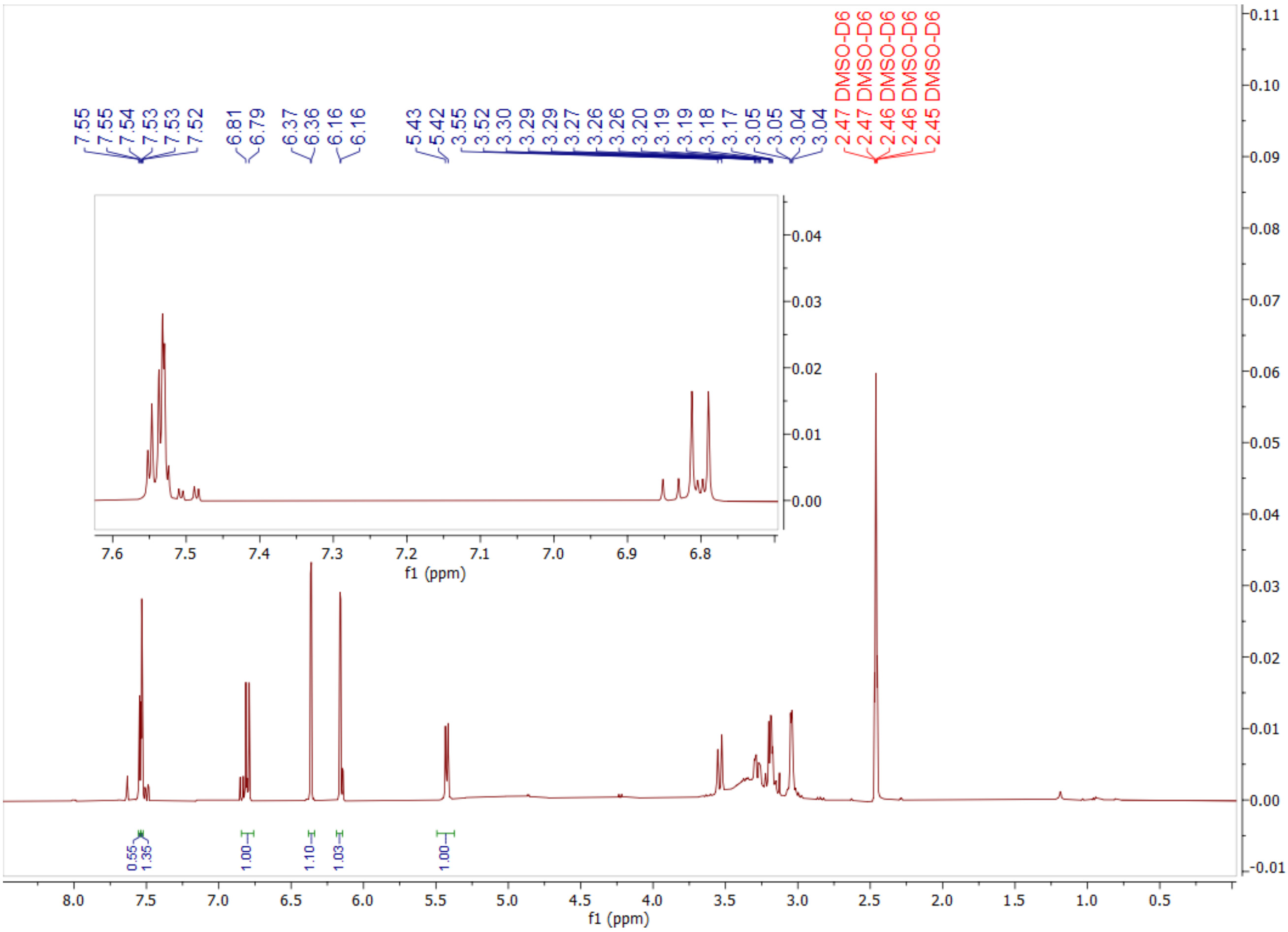

Figure S1. ^{1}H-NMR spectrum of Isoquercitrin (400 MHz, DMSO-*D6*).

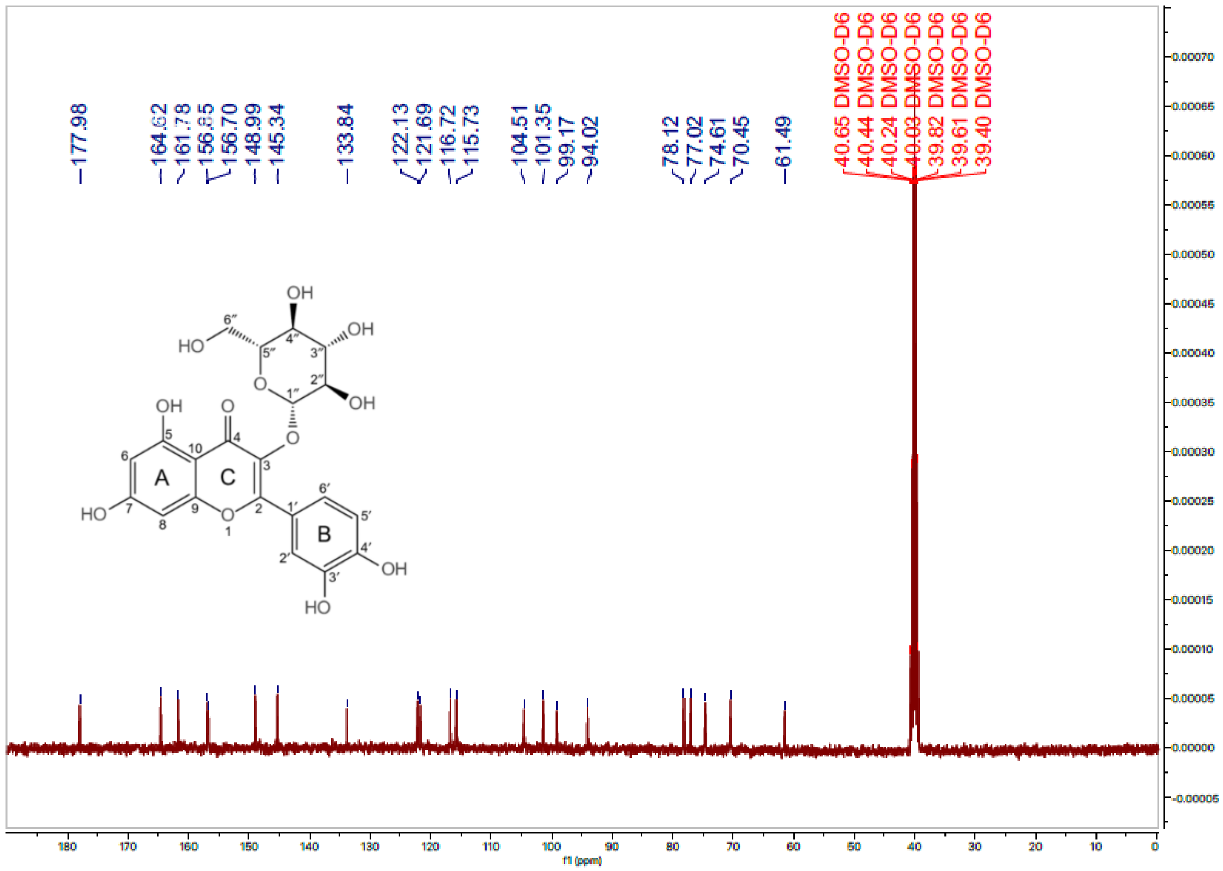

Figure S2. ^{13}C-NMR spectrum of Isoquercitrin (100 MHz, DMSO-*D*6)
